# Guided-Mode Resonance Grating with Self-Assembled Silver Nanoparticles for Surface-Enhanced Raman Scattering Spectroscopy

**DOI:** 10.3390/photonics1040380

**Published:** 2014-10-23

**Authors:** Jing Yang, Fanghui Ren, Xinyuan Chong, Donglei Fan, Swapnajit Chakravarty, Zheng Wang, Ray T. Chen, Alan X. Wang

**Affiliations:** 1 School of Electrical Engineering and Computer Science, Oregon State University, Corvallis, OR 97331, USA; 2 Department of Mechanical Engineering, The University of Texas at Austin, Austin, TX 78712, USA; dfan@austin.utexas.edu; 3 Omega Optics, Inc., Austin, Texas 78757, USA; swapnajit.chakravarty@omegaoptics.com; 4 Department of Electrical and Computer Engineering, University of Texas at Austin, Austin, TX 78758, USA

**Keywords:** surface-enhanced Raman scattering, grating, silver nanoparticles, self-assembling, guided-mode resonance

## Abstract

We designed and fabricated guided-mode resonance (GMR) gratings on indium-tin-oxide (ITO) thin film to generate a significantly enhanced local electric field for surface-enhanced Raman scattering (SERS) spectroscopy. Ag nanoparticles (NPs) were self-assembled onto the surface of the grating, which can provide a large amount of “hot-spots” for SERS sensing. The ITO gratings also exhibit excellent tolerance to fabrication deviations due to the large refractive index contrast of the ITO grating. Quantitative experimental results of 5,5’-dithiobis(2-nitrobenzoic acid) (DTNB) demonstrate the best enhancement factor of ~14× on ITO gratings when compared with Ag NPs on a flat ITO film, and the limit of detection (LOD) of DTNB is as low as 10 pM.

## 1. Introduction

Surface-enhanced Raman scattering (SERS) is a powerful technique that can provide ultra-sensitive and non-destructive characterization of various kinds of molecules [1]. Single molecule-level detection has been achieved with enhancement factors (EFs) as large as 10^14^–10^15^ from the “hot spots” of noble metallic nanostructures [2–7]. However, the density of such “hot spots” is usually extremely low, and this limits the “average” enhancement factor of SERS substrates at a large scale. Therefore, a comprehensive enhancement mechanism that can produce a universal increase of the SERS signals across the entire substrate is highly desirable for SERS sensing. Sub-wavelength plasmonic grating structures have drawn intensive interest since the discovery of extraordinary optical transmission through metal films with hole arrays discovered by Ebbesen *et al.* in 1998 [8]. Recently, numerous approaches based on periodic arrays have been utilized as SERS substrates with high sensitivity and selectivity, due to their unique optical resonance features and the strong confinement to the electromagnetic field [9,10]. On the other hand, guided-mode resonance (GMR) in dielectric gratings has also been discovered to enhance the localized surface plasmon polaritons (LSPPs) of plasmonic NPs, which will further increase the SERS signals [11–14]. The working mechanism relies on resonant modes when properly excited, which can serve as a universal field-enhancement substrate. Our previous study demonstrated a SERS substrate by coating Si_3_N_4_ gratings with plasmonic active SiO_2_ nanotubes, and the substrate showed a constant enhancement factor of 8~10 in addition to the existing SERS effect of SiO_2_ nanotubes [15]. However, in such devices, the Ag NP-coated SiO_2_ nanotubes were sparsely distributed on the surface of gratings, which only provided a limited number of “hot-spots” on a small area of the GMR gratings. We also demonstrated SERS sensing using bio-enabled photonic crystal structures, in which a monolayer of Ag NPs were self-assembled on diatom frustules [16,17]. In these studies, the LSPs of Ag NPs was coupled with the GMRs of diatom frustules, which provided stable SERS enhancement in addition to the existing SERS effect of Ag NPs. However, due to the variation of the shape and dimension of the naturally-obtained diatom frustules, the SERS enhancement of diatom-based substrates is not as high as rationally designed gratings.

In this work, we design and fabricate a GMR grating on ITO thin film with resonant wavelength at 532 nm. The essential idea towards high sensitivity SERS detection is the engineering of the GMR grating, which can produce a universal increase of the SERS signal intensity across the entire substrate with a high density of hot-spots. A 100 nm-thick spin-on glass (SOG) is coated on top of the ITO grating. This thin layer of SOG will prevent direct contact of Ag NPs with the ITO grating to avoid the strong damping effect, while still achieving reasonable coupling efficiency between the Ag NPs and ITO grating. The Ag NPs are self-assembled onto the SOG layer to provide a sufficient amount of “hot-spots” for SERS enhancement. In addition, the ITO grating exhibits great tolerance to fabrication deviations, due to the strong refractive index contrast between the ITO and the SOG. Our ITO-based SERS substrates demonstrate significant Raman enhancement capability, and the quantitative SERS characterization of DTNB on gratings showed that the LOD of the ITO gratings is 10 pM.

## 2. Experimental Section

### 2.1. Reagents and Materials

DTNB, ethylenediaminetetraacetic acid, and poly-diallyldimethylammonium chloride (PDDA) were purchased from Sigma-Aldrich. Silver nitrate was purchased from Alfa Aesar. Trisodium citrate was purchased from Macron chemicals. Isopropanol and acetone were obtained from the chemistry store at Oregon State University. ITO substrates were purchased from Delta Technologies, Ltd. All chemicals and materials used were analytical grade and used as received. Deionized (DI) water (18.1ΩM *cm was used throughout the experiment.

### 2.2. ITO GMR Grating Fabrication

**Figure 1:**
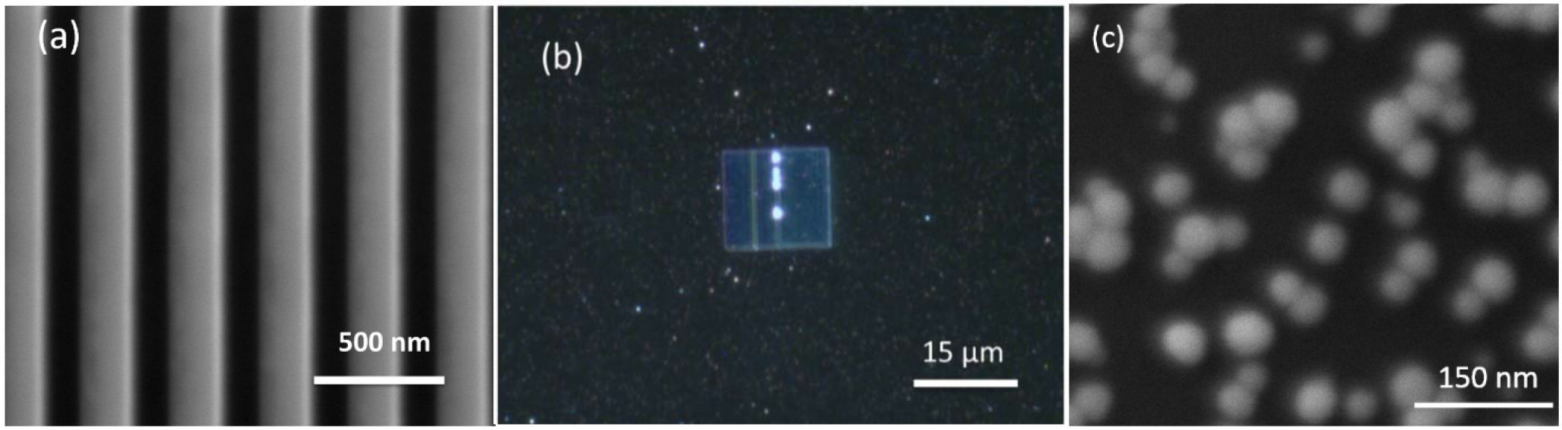
(**a**) SEM image of the fabricated ITO grating structure after focused ion beam (FIB) patterning; (**b**) optical image of the Ag NP self-assembled grating; and (**c**) SEM image of the Ag NPs.

The device fabrication started with ITO-coated glass substrates. The ITO-on-glass substrates were cleaned by acetone, followed by rinsing successively with isopropanol and deionized water. The substrates were dried with high-purity nitrogen. The thickness of the ITO layer is 200 nm with a surface resistivity of 5 Ω/square. A grating structure with an area of 15 μm × 15 μm was patterned by a focused ion beam (FIB), with a gallium ion current of 50 pA and a dose of 15 mC/cm^2^. The periodicity of the grating was controlled at 336 nm with gap widths of 150 nm. A scanning electron microscopy (SEM) image of the grating structure after the FIB milling is shown in Figure 1a. A thin layer (~100 nm) of SOG was used to cover the ITO grating at a spinning speed of 5000 rpm for 30 s. The device was baked at 220 °C in order to remove the SOG solvent.

### 2.3. Silver Nanoparticle Synthesis and Self-Assembling

Ag NPs were synthesized according to the method developed by Stokes *et al.* [18]. The Ag NPs we synthesized are uniform and are 40–50 nm in diameter. The ITO grating was self-assembled with Ag NPs to serve as the SERS-active substrate. In brief, the ITO grating was first functionalized with 1% PDDA, a cationic polyelectrolytes, for 30 min. After three times rinsing with deionized (DI) water, the PDDA functionalized ITO grating was immersed into ethylenediaminetetraacetic acid-reduced Ag NPs for 1 h. The Ag NPs were then self-assembled onto the surface of the positive-charge-modified ITO gratings by electrostatic interactions. The optical image of the Ag NPs functionalized the ITO grating, and the SEM images of the Ag NPs are shown in Figure 1b,c, respectively. The vertical lines in Figure 1b are due the FIB defects. The slit widths of those two lines are different compared with other slits, due to the stiches of different regions during the ion beam writing process. The four spots are bigger nanoparticle clusters, which are brighter compared with the nanoparticles on the glass substrates, due to the enhancement from grating. In our measurement, we intentionally avoided those regions, so the defects will not play any role in SERS characterization. The devices were then rinsed with DI water three times for future SERS test.

### 2.4. Instrumentation

To acquire SERS spectra from the Ag NPs on ITO gratings and on flat ITO thin film, 10 μL of DTNB aqueous solution with different concentrations was dropped onto the surface of ITO gratings, as well as the surrounding areas. SERS measurements were acquired with a Horiba Jobin Yvon Lab Ram HR 800 Raman microscope. All samples were excited with a 1.2-mW, 532-nm excitation laser through a 10× objective lens, which was manually focused to obtain the highest Raman signals. The confocal pinhole was set to a diameter of 100 μm, which minimized the background signals and improved the signal-to-noise ratio. Raman light was collected by a CCD camera with a 1200-line mm^−1^ grating. Raman spectra were acquired with a 10-s integration time in the Raman spectral range from 400 cm^−1^ to 2000 cm^−1^. Spectra were recorded and processed with Horiba LabSpec.5 data acquisition and analysis software, which was used for spectra baseline subtraction.

## 3. Results and Discussion

### 3.1. Design and Characterization of the Medium Q-Factor ITO Grating

**Figure 2:**
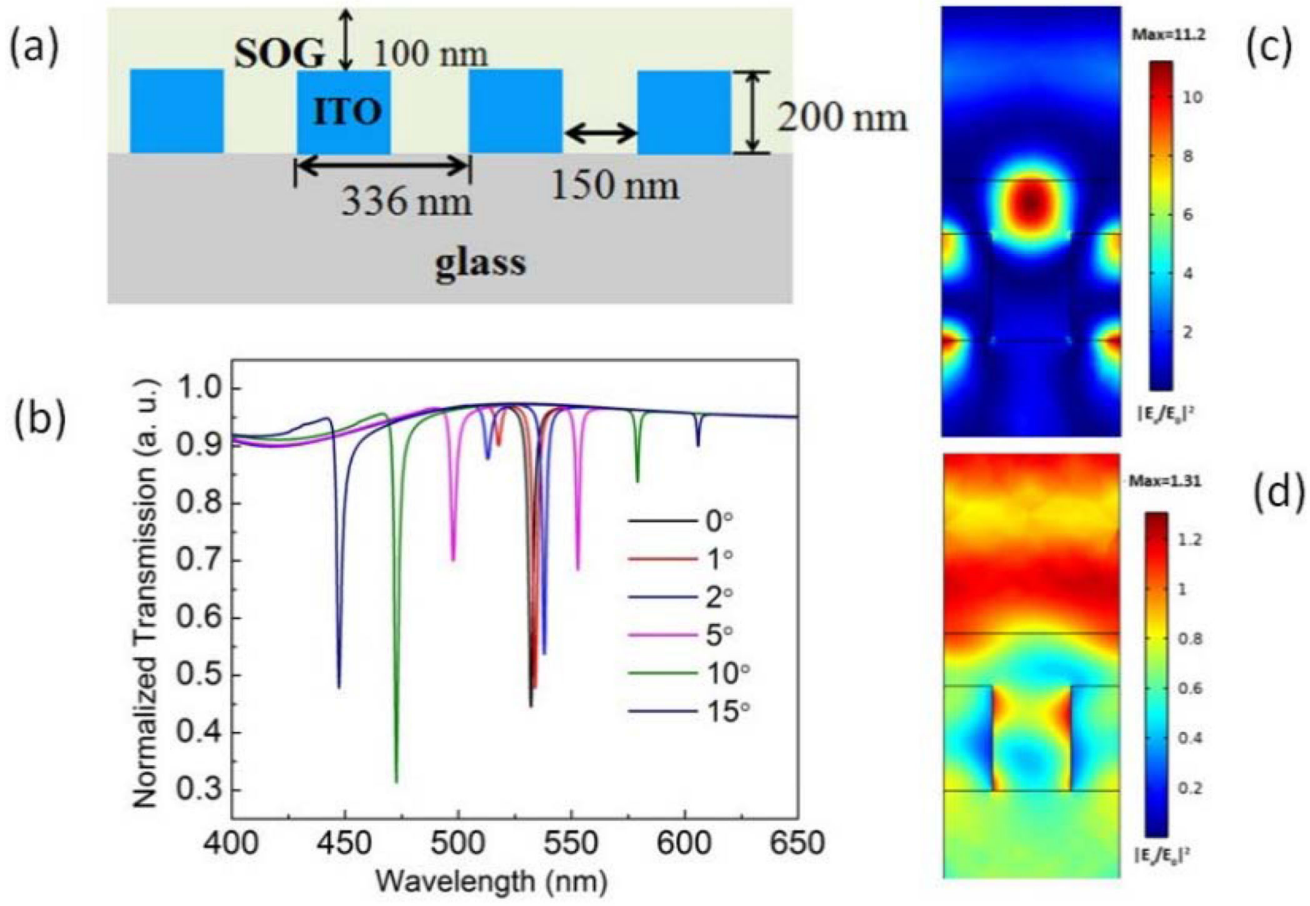
Schematic of the proposed guided-mode resonance (GMR) grating (**a**); the simulated optical transmission spectra of the ITO grating with different incident angles (**b**); and the electric field *Ex* distribution in the X–Z plane with a normal incident angle (**c**) and a 5° incident angle (**d**). SOG, spin-on glass.

We used DiffractMod module in RSoft, which is based on rigorous couple wave analysis (RCWA), to design the ITO gratings. The RCWA method can provide a rapid solution of the eigenmodes supported by the grating. The GMR of the ITO grating is formed by the optical coupling between the discrete guided modes of the photonic crystal slab and the radiation continuum above the light line. The permittivity of the ITO layer was calculated using the Drude model, which is a classical free-electron theory to model the dielectric permittivity of transparent conductive oxides [19]. At the 532-nm wavelength, the permittivity of ITO was calculated to be 3.364 + 0.029 i, which is a dielectric material with a small imaginary part. The schematic of the ITO grating is shown in Figure 2a. In our simulation, plane waves with different incident angles were used as the excitation light, and the transmission spectra of the ITO grating are shown in Figure 2b. The magnetic field of the light is polarized perpendicular to the incident plane, which is termed the transverse-magnetic (TM) wave. It can be seen that at normal incidence, the transmission curve has a single dip with a line width of ~3 nm, indicating a relatively high Q-factor above 150. When the incident angle increases, the single dip splits into two dips, and the deviation is nearly proportional to the incident angle. This proves that if the incident light is not perfectly collimated, the experimentally-measured Q-factor can be much lower. Figure 2c,d shows the electric fields distribution in the x–z plane with a normal and 5° incident wave at the 532-nm wavelength. The maximum electric field enhancement factor |*E_x_/E_0_*|^*2*^ (where *E_0_* is the electric field amplitude of the incident light) reaches more than 11 and only 1.3, respectively. In addition, we can see that the peak electric field in Figure 2c is close to the SOG/air interface with a strong evanescent field, which should be beneficial to the optical coupling with Ag NPs. While for the 5° incident light in Figure 2d, the field enhancement is negligible.

**Figure 3:**
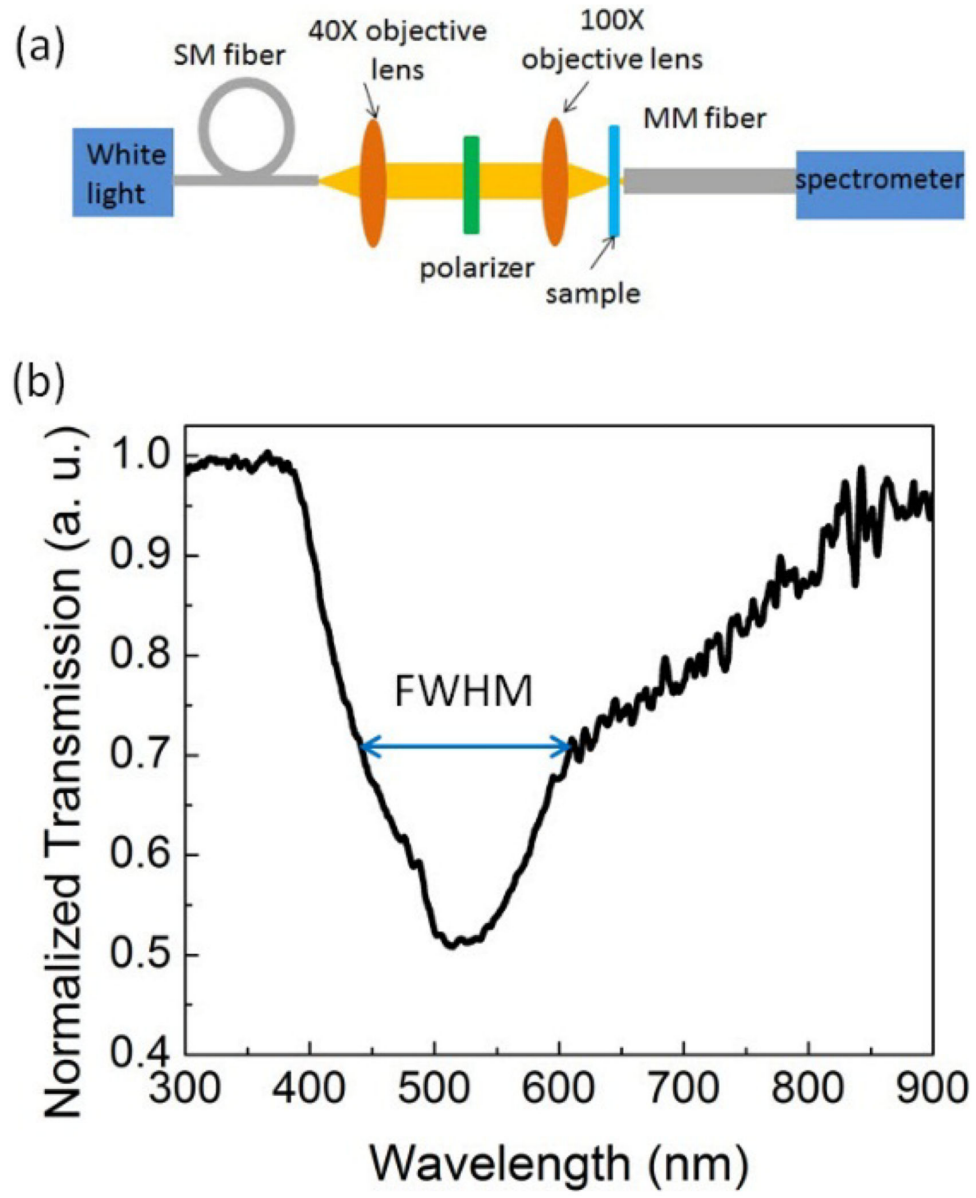
(**a**) Experimental setup of the transmission measurement; and (**b**) the experimentally measured transmission spectrum of the ITO grating coated with SOG. SM, single mode.

The optical transmission of the ITO gratings is experimentally measured. Figure 3a shows the experimental setup. A white light source (AvaLight-HAL, AVANTES Inc., Netherlands with a 300–900 nm wavelength was coupled into a single-mode (SM) polarization-maintaining fiber. The output light was then collimated by a 40× objective lens (NA = 0.65). The device was mounted at the focal plane of a 100× objective lens on an xyz stage. The transmitted beam after the sample was collected by a multimode fiber and coupled into a visible wavelength spectrometer (USB2000+, Ocean Optics, Inc., USA). Figure 3b shows the experimentally-measured transmission spectrum of the ITO grating coated with 100-nm SOG, which shows the extinction peak at 525 nm with a full width half maximum (FWHM) of ~180 nm (420–600 nm). The broadened resonance and the reduced Q-factor are mostly due to the focused light of the high numerical aperture lens.

### 3.2. Numerical Investigation of the Optical Coupling between Ag NPs and the ITO Grating

**Figure 4:**
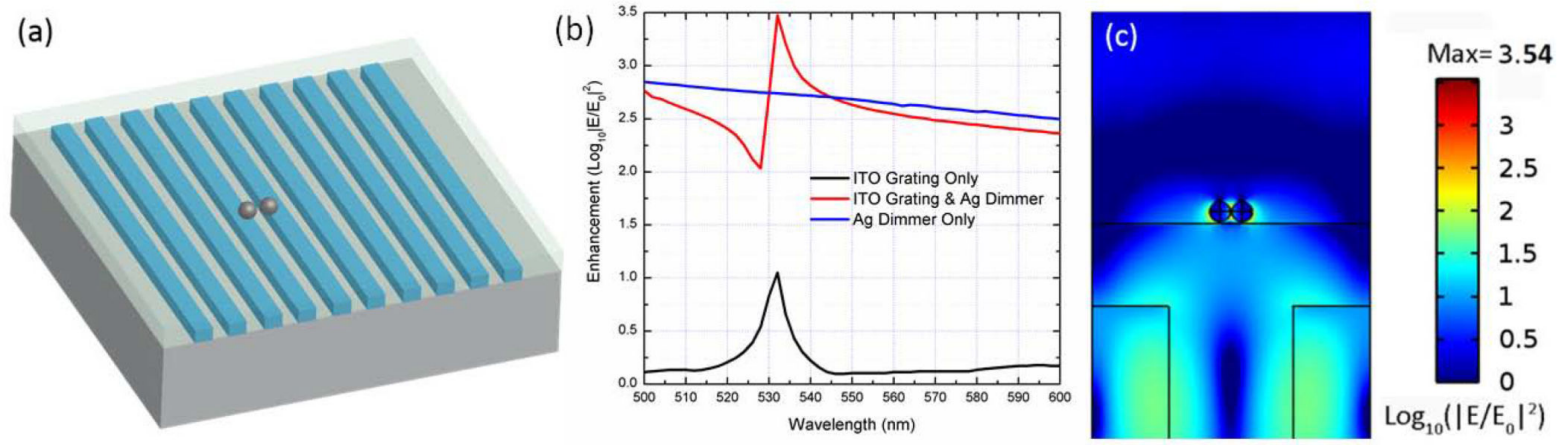
(**a**) 3D COMSOL model; (**b**) peak electric filed enhancement of three different surface-enhanced Raman scattering (SERS) substrates; (**c**) electric field distribution (side view) of the ITO grating with a Ag dimer on top of the SOG layer at 532-nm excitation.

To investigate the interaction between the GMR mode and the LSP of Ag NPs, we used the RF module from COMSOL 4.4 to numerically compare the electric field enhancement of three different SERS substrates: (1) an ITO grating without any Ag NP; (2) a Ag dimer on top of the SOG surface; and (3) an ITO grating with a Ag dimer on top of the SOG surface, which mimics the real experimental configuration. The diameter of the Ag NPs is 25 nm, and the gap between the NPs is 2 nm. As shown in the 3D model in Figure 4a, a TM plane wave is normally incident to the grating surface, as the simulation configuration in Figure 2. Figure 4b shows the peak electric field enhancement of the three SERS substrates as a function of wavelength. The Ag dimer on the flat SOG surface substrate has a very broad band resonance (almost flat) in the simulated wavelength window, while the other two substrates show resonance peaks at 532 nm. The enhancement factors |*E_x_/E_0_*|^*2*^ of the ITO grating are a typical Lorentzian resonance with a relatively low peak value (~11), while the Ag dimer on top of the SOG layer has a very distinctive Fano resonance with asymmetric enhancement factors, and the peak value reaches ~3000, which is about 5× higher than the Ag dimer on the flat SOG surface. The log-scale optical intensity distribution of the SERS substrate is shown in Figure 4c, which shows that the maximum field is located in the gap of the dimer. We can also clearly observe that the GMR of the ITO grating in Figure 4c is different than that in Figure 2c, since it is hybridized with the LSP of the Ag dimer. The hybridization of the broadband LSP with the narrow band GMR will lead to a distinctive Fano resonance, as shown in Figure 4b.

**Figure 5:**
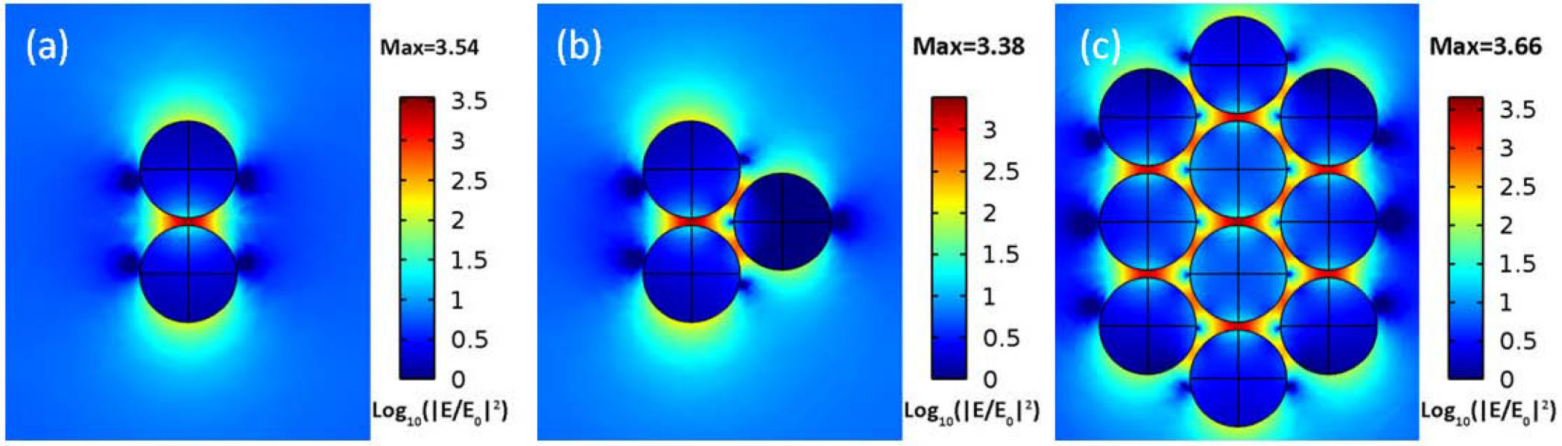
Electric field distribution (top view) of (**a**) a Ag dimer, (**b**) a Ag trimer and (**c**) a cluster with ten highly-packed Ag NPs on top of the SOG surface above the ITO grating at 532-nm excitation.

From the SEM image in Figure 1c, most of the Ag NPs agglomerate together to form dimers, trimers or even larger clusters. Therefore, it is necessary to study the effect of the number of the NPs. In the simulation in Figure 5a–c, we choose three typical nanostructures: a dimer, a trimer and a big cluster with ten highly-packed NPs. As expected, the maximum electric fields are all located in the gap of the NPs, and more NPs create more “hot spots”. The peak electric field enhancement factor is relatively uniform as the number of NPs increases.

### 3.3. SERS Sensing with the Ag NPs on ITO Gratings

**Figure 6:**
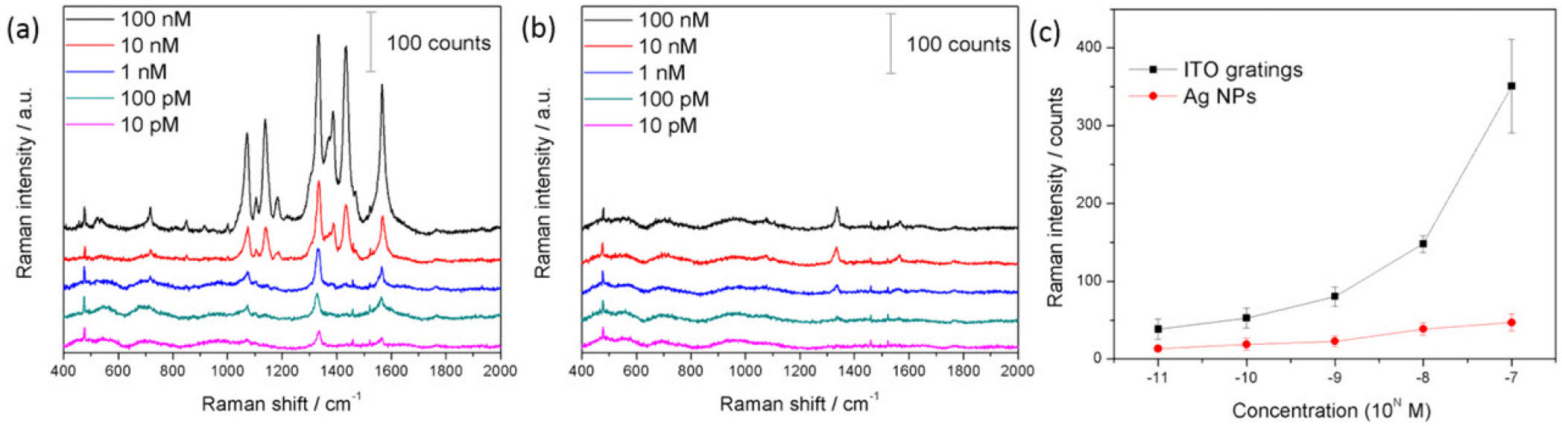
SERS spectra of 5,5’-dithiobis(2-nitrobenzoic acid) (DTNB) with concentrations ranging from 100 nM to 10 pM on (**a**) ITO gratings and on (**b**) bare Ag NPs; (**c**) a comparison of the Raman peaks at 1331 cm^−1^.

The ITO gratings with self-assembled Ag NPs were exploited for SERS detection. In this study, we utilized DTNB, a widely-used Raman label molecules, as the Raman reporter. To quantitatively investigate the SERS signals, the DTNB aqueous solution (10 μL) with different concentrations ranging from 100 nM to 10 pM was dropped onto the surface of the Ag NPs on ITO gratings. The Raman signals of DTNB solution were collected by a confocal Raman spectrometer with the excitation laser at 532 nm. Each spectrum represents the average measurement results of 10 randomly-selected spots on the ITO gratings. As shown in Figure 6a, DTNB exhibits distinctive Raman characteristic bands at 716 cm^−1^, 1070 cm^−1^, 1138 cm^−1^, 1186 cm^−1^, 1331 cm^−1^, 1389 cm^−1^, 1432 cm^−1^ and 1564 cm^−1^. The most prominent Raman peak at 1331 cm^−1^ is assigned to the symmetric stretch of the nitro group of DTNB [20]. The Raman signals of DTNB increase as the concentration increases. Pronounced SERS signals are observed even at a 10-pM DTNB concentration. We compared the SERS signals of DTNB from Ag NPs on ITO gratings and from Ag NPs on flat film by collecting the SERS signals on the ITO gratings and on the surrounding areas of ITO gratings. As shown in Figure 6b, the SERS signals of DTNB on flat films are much weaker than those on ITO gratings. A more quantitative analysis of the SERS signals of DTNB is presented in Figure 6c. These two curves were obtained by plotting the Raman peak of DTNB at 1331 cm^−1^ with respect to different concentrations of DTNB solution from Ag NPs on ITO gratings and outside ITO gratings, as indicated by the black and red curves, respectively. For the prominent Raman signature peak of DTNB at 1331 cm^−1^, the Raman signal intensity on the ITO gratings is enhanced by 3–14-times when compared with that on the area without gratings. In addition, the ITO gratings show higher enhancement factors at higher concentrations than at lower concentrations.

**Figure 7:**
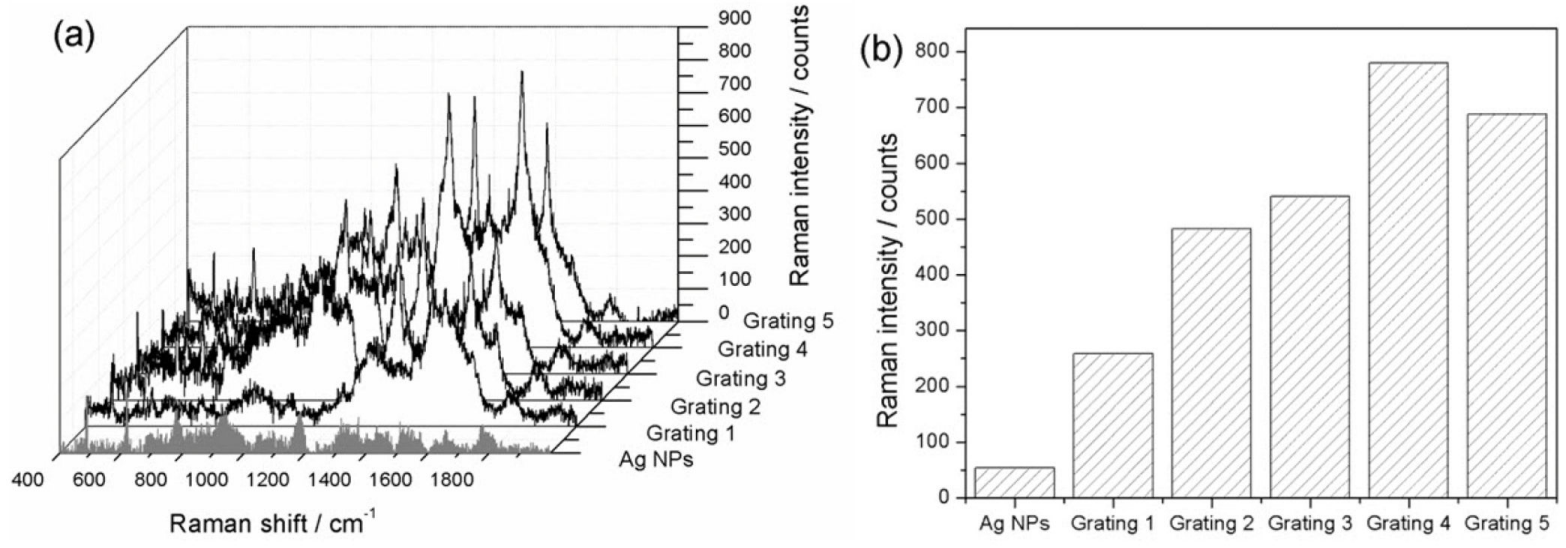
SERS performance of 1 μM DTNB on different gratings (**a**) and Raman intensities of the band at 1331 cm^−1^ of DTNB from different gratings (**b**).

The relationship between the SERS enhancement and the fabrication variation of the ITO gratings were also investigated. Figure 7a shows the SERS signals of DTNB (1 μM) of Ag NPs on the flat film and on five different ITO gratings with periodicities of 325 nm, 330 nm, 335 nm, 340 nm and 345 nm. Figure 7 shows the DTNB Raman signal intensities of the peak at 1331 cm^−1^ on flat film and on the five gratings. When compared with the flat film substrate, all of the five ITO gratings showed significant SERS enhancement with different enhancement factors ranging from 5 to 14, as shown in Figure 7b. The results indicate that the fabrication tolerance of our ITO gratings is very high, due to the broadened resonant peak of the ITO grating.

## 4. Conclusions

In conclusion, we have numerically investigated and experimentally demonstrated a SERS substrate comprised of an ITO GMR grating with plasmonic-active Ag NPs. The Ag NPs can provide sufficient “hot-spots” for SERS enhancement and also achieve sufficient LSPs. Our ITO gratings also demonstrate great tolerance to the geometry variation of the devices. Quantitative experimental measurement of DTNB confirms that the SERS substrate can provide the best enhancement factor of ~14× across the entire grating surface in addition to the existing SERS effect of Ag NPs. As a new enhancement mechanism to SERS technology, the GMR grating-coupled Ag NPs demonstrated in this paper proves that resonant photonic devices can indeed serve as an effective substrate to increase the sensitivity of Raman spectroscopy.
